# Carotid Atherosclerotic Plaque Matrix Metalloproteinase-12–Positive Macrophage Subpopulation Predicts Adverse Outcome After Endarterectomy

**DOI:** 10.1161/JAHA.112.001040

**Published:** 2012-12-19

**Authors:** Vincent P. W. Scholtes, Jason L. Johnson, Nicholas Jenkins, Graciela B. Sala-Newby, Jean-Paul P. M. de Vries, Gert Jan de Borst, Dominique P. V. de Kleijn, Frans L. Moll, Gerard Pasterkamp, Andrew C. Newby

**Affiliations:** Department of Vascular Surgery, UMC Utrecht, Utrecht, the Netherlands (G.J.d.B., F.L.M.); Laboratory Experimental Cardiology, UMC Utrecht, Utrecht, the Netherlands (V.P.W.S., D.P.V.d.K., G.P.); Bristol Heart Institute, University of Bristol, Bristol, United Kingdom (J.L.J., N.J., G.B.S.-N., A.C.N.); Department of Vascular Surgery, St Antonius Hospital, Nieuwegein, the Netherlands (J.-P.P.M.d.V.)

**Keywords:** atherosclerosis, macrophages, MMP-12, outcome

## Abstract

**Background:**

Matrix metalloproteinase-12 (MMP-12) promotes atherosclerosis in animal models. MMP-12 is expressed in only a subset of foam-cell macrophages (FCMs) in human plaques. We investigated whether the prevalence of this MMP-12–expressing subpopulation is a prognostic indicator of adverse outcome in patients after carotid endarterectomy (CEA).

**Methods and Results:**

Serial sections of culprit lesions from 236 patients who underwent CEA and had undergone 3 years of clinical follow-up were stained immunocytochemically for MMP-12 and for CD68, and the MMP-12/CD68 ratio was used to quantify the MMP-12–expressing subpopulation. A high MMP-12/CD68 ratio correlated with a high content of lipid and total macrophages and a low content of vascular smooth muscle cells, as well as with MMP-8 (*R*=0.211, *P*=0.001), MMP-9 (*R*=0.251, *P*<0.001), and cleaved caspase-3 (*R*=0.142, *P*=0.036) activity measured in a neighboring segment. Dual immunohistochemical examination confirmed the location of MMP-12 in a subpopulation of MMP-8– and MMP-9–positive FCMs, whereas all apoptotic FCMs were MMP-12 positive. Patients who yielded plaques within the highest quartile compared with the lowest quartile of MMP-12/CD68 ratio had a 2.4-fold (hazard ratio, 2.4; 95% CI, 1.1- to 5.1-fold; adjusted *P*=0.027) increased risk of major adverse cardiovascular event and a 3.4-fold (3.4; 1.2- to 9.6-fold, *P*=0.024) increased risk for stroke.

**Conclusions:**

The prevalence of an MMP-12–positive subset of FCMs is a prognostic marker for adverse clinical outcome after CEA.

## Introduction

Thrombosis due to atherosclerotic plaque rupture remains the leading cause of death in Western countries,^1^ despite currently applied therapies. Furthermore, patients who have experienced an atherothrombotic event are at increased risk for another, either in the same territory or remotely.^2,3^ Matrix metalloproteinases (MMPs) are a family of Zn^2+^-containing proteinases that are believed to play a direct role in plaque development and rupture.^4^ Several MMPs promote an unstable plaque phenotype by destroying the extracellular matrix of the fibrous cap and facilitating penetration, proliferation, and apoptosis of inflammatory cells including macrophages.^5–7^ MMP-12 overexpression, in particular, accelerates lesion development in transgenic rabbits^8,9^; 2 studies in MMP-12 knockout mice demonstrated reduced plaque growth and beneficial effects on histological surrogates of plaque instability.^5,10^ Furthermore, a selective MMP-12 inhibitor halts atherosclerosis development and promotes a more stable plaque phenotype in mice.^11^ MMP-12 is expressed in a subpopulation of foam-cell macrophages (FCMs) located around the lipid core in human and rabbit plaques.^12–14^ Several distinct subpopulations of FCMs are found in plaques,^15^ of which one was based on staining positive for MMP-14 and negative for tissue inhibitor of metalloproteinase-3.^16^ Some of these subpopulations are thought to promote plaque instability, whereas others may be atheroprotective.^15^ However, it is not been shown previously whether the occurrence of any of these subpopulations is predictive of adverse outcome.

Our group has pioneered the concept that some biochemical and cellular characteristics of culprit plaques carry predictive value for future events.^17–19^ Of relevance, we previously showed that tissue levels of MMP-8 and MMP-9 in culprit plaques retrieved after carotid endarterectomy (CEA) are associated with increased risk of major adverse cardiovascular events (MACEs) during clinical follow-up, whereas MMP-2 levels predicted reduced risk.^20,21^ This study investigated whether prevalence of the MMP-12–positive FCM subpopulation predicts clinical outcome after endarterectomy.

## Methods

### Patients and Plaques

Plaques were obtained from patients who underwent carotid endarterectomy (CEA) and were part of the Athero-Express Biobank.^22^ Culprit lesions from 236 patients were chosen from 499 available patients who had undergone CEA within 2002–2006 and who had known follow-up and measurements of MMP-2, MMP-8, MMP-9, and cleaved caspase-3 activity levels. The cohort consisted of 236 randomly selected patients. To increase the event rate, we have inserted extra cases, resulting into a total of 55 cases and 181 controls. A case was defined as a person who had experienced a major adverse cardiovascular event (MACE).

### Follow-Up and Clinical Outcome

All patients had undergone a 3-year follow-up after CEA, including yearly visits to the outpatient clinic and duplex ultrasound investigations. Patients completed a questionnaire annually informing whether they had experienced any vascular event or had been hospitalized in the past year at 1, 2, and 3 years after surgery. Positive answers were validated by further inquiries according to a standard scheme; discharge letters and, if needed, laboratory measurements and results of additional studies, such as electrocardiograms or imaging studies, were collected from the institution where the potential event occurred. If patients did not respond to the follow-up questionnaire, their general practitioner was contacted. Each outcome event was agreed on by consensus of 2 members of the Outcome Assessment Committee or by the majority decision of 3 members.

### Definition of Outcome

The primary outcome was defined as any MACE. This included any cardiovascular-related death and nonfatal myocardial infarction or stroke. In addition, a subgroup was distinguished for any recurrent stroke.

### Histological and Biochemical Measurements

The study design and the standardized protocol of the Athero-Express Biobank with respect to plaque processing have been reported previously.^18,22^ The segment with the greatest plaque burden was considered to be the culprit lesion and subjected to histological examination by staining the section for CD68, smooth muscle actin (SMA), Picro Sirius Red (PSR), and hematoxylin-eosin (H-E). Macrophage and smooth muscle cell infiltration were quantitatively scored using computerized analyses (AnalySIS 3.2, Soft Imaging Systems GmbH, Munster, Germany) as well as semiquantitatively as “no,” “minor,” “moderate,” or “heavy.” The amount of collagen (PSR) and calcification (H-E) were semiquantitatively scored as “no,” “minor,” “moderate,” or “heavy” staining. Collagen content was estimated from the PSR sections with polarized light. The size of the lipid core was visually estimated at ×40 magnification. The lipid core size was analyzed in the H-E section, expressed as the percentage of total plaque area, and scored in 3 categories: “no fat,” “<40%,” and “>40%” of the total plaque area. The lipid core consisted of predominantly cholesterol clefts. Plaque hemorrhage was defined as the composite of plaque bleeding at the luminal side of the plaque as a result of plaque disruption and intraplaque hemorrhage, which is observed as a hemorrhage within the tissue of the plaque. Plaque hemorrhage was examined in H-E– and fibrin-stained samples and rated as being absent or present. Intraplaque vessels were stained with a CD34 antibody. Plaque vessel density was determined by the average number of vessels of 3 hotspots within every single plaque. A hot spot was defined as 1 high-power field at ×40 magnification. For vessel quantification, we used a grid (100×100 μm) overlying every hot spot to improve the reproducibility and to avoid counting vessels twice. The vessel density was determined by counting the number of vessels crossed by a bar of the grid within the selected hot spots. Increased vessel density was defined as an average vessel count per hot spot higher than the median of the cohort. The histological examination was performed by one senior analyst, who was blinded for the study of MMP-12–positive macrophages and clinical outcome.

MMP-2, MMP-8, MMP-9, and cleaved caspase-3 activity levels were determined in proteins isolated from the segment adjacent to the culprit lesion, as previously reported.^20,21^ Total activity of MMP-2, MMP-8, and MMP-9 was determined with use of Biotrak activity assays RPN2631, RPN2635, and RPN2634, respectively (Amersham Biosciences, Buckinghamshire, UK). To assess levels of apoptosis within atherosclerotic lesions, activity of cleaved caspase-3 was determined with luminescent assay (Promega Corporation, Madison, Wisc).

### Histological Staining Procedure and Image Analysis to Determine the Proportion of MMP-12–Positive Macrophages

Two adjacent sections, taken from the culprit lesion, were immunohistochemically stained for macrophages (CD68) and MMP-12, by using the following protocol:

Sections were dewaxed, by using Clearane (Leica Biosystems, Peterborough, UK) and rehydrated by using graduated concentrations of ethanol. Endogenous peroxidase activity was inhibited by incubating the sections with 0.3% H_2_O_2_ for 7 minutes. After rinsing, sections were heated in 10 mmol/L citrate buffer (pH 6.0) for 2 periods of 6 minutes. Sections were first incubated for 30 minutes with 10% goat serum. Second, sections were incubated overnight in a cold room with the primary antibody: CD68 mouse anti-human (DAKO, Glostrup, Denmark) and MMP-12 rabbit anti-human antibody (Abcam, Cambridge, UK). After overnight incubation, sections were washed and incubated with an appropriate biotinylated secondary antibody: CD68 secondary antibody: goat anti-mouse (DAKO) or MMP-12 secondary antibody: goat anti-rabbit (DAKO). Sections were incubated with extravidin–horseradish peroxidase (Sigma Aldrich, St Louis, MO) and stained with 3,3′-diaminobenzidine (Sigma Aldrich). Finally, sections were counterstained with hematoxylin.

### Procedure to Quantify the Amount of MMP-12–Positive FCMs

#### Semiautomated digital quantification

To quantify the presence of MMP-12–positive macrophages within a relative large amount of samples, we developed a new, semiautomated method. Sections were digitally scanned by using a digital scanner (ScanScope XT, Aperio, Vista, CA, USA). The scanning, storage, and data processing processes have been described in detail.^23^ Pictures were made by using the Aperio software program (Scanscope, Aperio). First, 3 snapshots of CD68-rich areas were taken from the CD68-stained section, by using a defined rectangle (Video S1), while blind to the MMP-12 staining. Second, snapshots were taken of the same area of the MMP-12–stained section (Video S2). Thirty-seven of the 236 patients did not have a correct scanned slide, due to problems with focus. Therefore, we used a light microscope (Olympus UK Ltd, Soutend-On-Sea, UK) and took snapshots of the atherosclerotic plaque in the same manner. The amount of positive surface area was quantified for each snapshot by using Cell P digital analysis software (version 2.8, Olympus Soft Images Solutions GbmH). Since only foamy macrophages stained for MMP-12, the ratio of foam cells that were positive for MMP-12 was calculated by dividing the total amount of positive area of the MMP-12 section with the total amount of positive area in the CD 68 section and multiplying by 100. Interobserver and intraobserver scores were good; the differences between the 2 measurements were within the limits of agreement, as reported previously by Bland and Altman.^24^ Analysis was performed by one researcher (V.S.). Sections that were damaged (5 sections) or not stained properly (3 sections) were excluded for analysis.

#### Manual quantification

To verify this semiautomated technique we used a more laborious individual cell counting procedure in a subgroup of 62 samples. All MMP-12–positive cells were also positive for CD68 and appeared foamy. Hence, all the MMP-12–positive cells were counted in the first section and all the CD68-positive cells were counted in the second section, and the ratio was calculated and multiplied by 100 to give percentage positivity of CD68 cells for MMP-12. All analyses were performed by a single observer (N.J.).

### Dual Immunohistochemistry and Image Analysis

Three adjacent sections, taken from the culprit lesion, were immunohistochemically stained with primary antibodies for MMP-12 and either cleaved caspase-3 (goat anti-human; R&D Systems, Abingdon, UK), MMP-8 (mouse anti-human; Merck Millipore, Watford, UK), or MMP-9 (mouse anti-human; Merck Millipore). Fluorophore conjugated secondary antibodies were used to yield either a red fluorescent product at the site of the antigen (Alexafluor 594 for MMP-12) or a green fluorescent product at the site of the antigen (Alexafluor 488 for cleaved caspase-3, MMP-8, and MMP-9). Sections were then mounted in ProLong Gold Antifade Reagent with 4′,6-diamidino-2-phenylindole (Life Technologies, Paisley, UK; P-36931) to fluorescently label nuclei blue. The specificity of the immunolabeling was demonstrated by inclusion of a negative control using isotype-specific nonimmune serum or IgG. The percentage of MMP-12–positive macrophages that stained for other proteins was counted within each lesion (n=15).

### Statistics and Data Analysis

For statistical analyses, PASW statistics 17.0 was used (SPSS Inc, Chicago, Ill). The percentage of MMP-12–positive macrophages was not normally distributed. Clinical and histological plaque characteristics were analyzed by using a Mann–Whitney *U* test (2 groups) or Kruskal–Wallis test (≥3 groups). Continuous variables were analyzed by using Spearman's rank correlation test. Ordinal values versus ordinal values were analyzed with an ordinal regression analysis. Survival analysis was performed by using a Cox regression analysis including MMP-12 quartiles and traditional risk factors that had been demonstrated to be significantly associated with outcome (*P*<0.05). Determinants with *P*<0.05 were regarded as statistically significant. The authors had full access to and take full responsibility for the integrity of the data. All authors have read and agree to the article as written.

## Results

### Quantification of the MMP-12–Positive FCM Subpopulation

**Figure 1.**
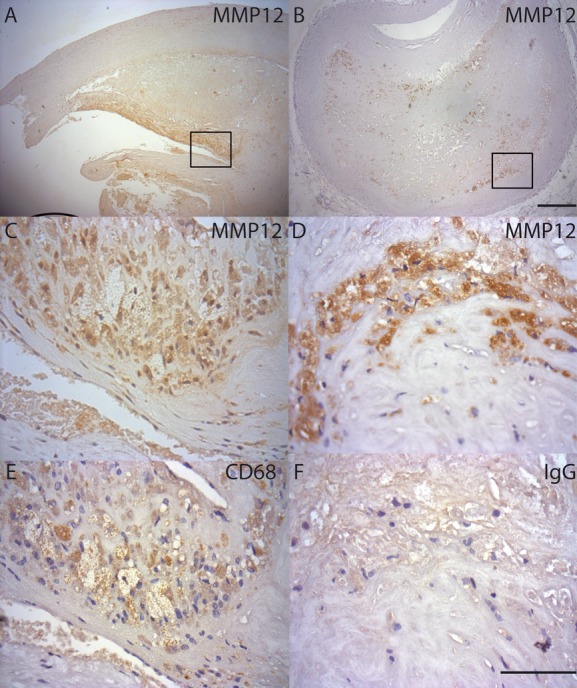
Immunocytochemistry for MMP-12 and CD68 in human atherosclerotic plaques. A, A plaque section stained with antibodies against MMP-12 (magnification ×40). B, A section of a different plaque stained as in A. C, The boxed area of A (magnification ×400). D, The boxed area of B (magnification ×400). E, The corresponding area in C in a serial section stained with anti-CD68. F, The corresponding area in D in a serial section stained with nonimmune IgG. The bar within A and B represents 1 mm, and the bar within C to F represents 0.1 mm.

**Figure 2.**
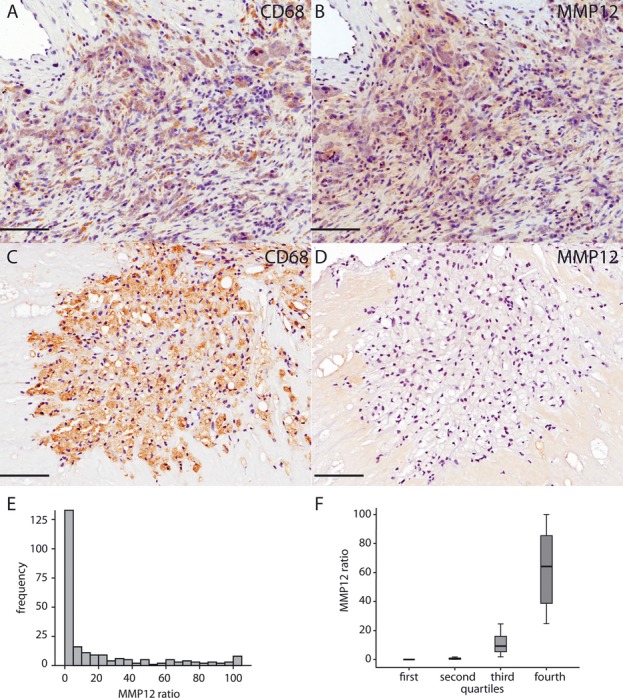
Comparison of MMP-12– and CD68-stained areas in CEA specimens. A, An example of a CD68-positive area of interest selected for analysis while blind to the MMP-12 staining. B, The serial section stained for MMP-12. Most CD68 cells are also MMP-12 positive. C, A different CD68-positive area of interest selected for analysis. D, The area in C stained for MMP-12 in a serial section. Most CD68-positive cells are negative for MMP-12. E, The distribution of MMP-12–positive macrophages across all the patients. F, A box-whisker plot showing the values of MMP-12 positivity in the 4 quartiles. The bar within A to D represents 0.2 mm.

Culprit lesions were obtained from 236 patients who underwent CEA, whose characteristics are summarized in Table 1, columns 1 and 2. Serial sections were immunostained for MMP-12 and CD68 (Figures 1 and 2). Nonimmune controls revealed that the staining was specific (Figure 1D compared with 1F). The majority of MMP-12–positive cells were located in deeper parts of the plaque surrounding the necrotic cores, in agreement with previous work (Figure 1A and 1B).^12^ In such areas, MMP-12 staining was located exclusively in CD68-positive cells with a foamy appearance, that is, in FCMs. Furthermore, whereas most CD68-positive FCMs around the core stained for MMP-12 (Figures 1C, 1D, and 2B), very few CD68-positive cells stained for MMP-12 in other plaque areas (Figure 2D). We concluded that MMP-12 was expressed only in a subpopulation of FCMs, which confirmed the results of previous studies.^12^

**Table 1.** Patient Characteristics and Their Relationship to the Percentage of MMP-12–Positive Macrophages

		MMP-12/CD68 ratio		
			
Characteristic	Total Cohort, n (%)	No	Yes	*P*
Age >69 y (median)		1.9 [0 to 29.0]	1.8 [0 to 22.9]	0.539
Male sex	178 (75.4)	0.4 [0 to 7.9]	4.4 [0 to 34.1]	*0.004*

Hypertension	176 (74.6)	0.5 [0 to 15.4]	2.6 [0 to 28.3]	0.127

Diabetes	40 (16.9)	1.8 [0 to 24.7]	5.2 [0 to 28.7]	0.645

Hypercholesterolemia	132 (55.9)	1.8 [0 to 18.4]	1.9 [0 to 31.7]	0.470

Smoking*	81 (34.3)*	3.3 [0 to 27.6]	1.0 [0 to 24.7]	0.452

Positive family history†	59 (26.9)†	1.2 [0 to 17.7]	5.5 [0 to 29.0]	0.173

History of myocardial infarction	30 (12.7)	1.8 [0 to 20.7]	19.4 [0.2 to 58.4]	*0.043*

History of coronary intervention	54 (22.9)	1.2 [0 to 20.7]	4.8 [0.2 to 59.0]	*0.031*

Previous myocardial infarction or coronary intervention	66 (28.0)	1.2 [0 to 18.0]	6.0 [0.2 to 53.3]	*0.017*

History of peripheral intervention	44 (18.6)	1.9 [0 to 24.5]	1.5 [0 to 33.2]	0.929

Previous stroke	27 (11.4)	1.8 [0 to 24.4]	1.8 [0 to 51.9]	0.952

Asymptomatic vs symptomatic	41(17.4) vs 195 (82.6)	1.0 [0 to 27.6]	1.9 [0 to 24.6]	0.445

Asymptomatic vs AFX	41(17.4) vs 29 (12.3)	1.0 [0 to 27.6]	4.1 [0 to 42.2]	0.778

Transient ischemic attack vs stroke	110 (46.6) vs 56 (23.7)	1.9 [0 to 25.1]	1.8 [0 to 24.1]	0.862

Median values and quartile 1 to quartile 3 (between brackets) of MMP-12 ratio are depicted for patients without or with a characteristic. AFX indicates amaurosis fugax.

* The smoking status of 2 patients could not be retrieved.

† The family history of 17 patients could not be retrieved.

To quantify the MMP-12–positive subpopulation, we selected 3 fields with abundant CD68 staining and measured the CD68-positive area while still blind to the MMP-12 staining (Figure 2A and 2C, Video S1). We then measured the area of MMP-12 staining in the corresponding fields of the serial section (Figure 2B and 2D, Video S2) and computed the MMP-12/CD68 ratio as a percentage. The average value for the 3 areas was assigned to each patient. To validate this semiautomated histological analysis, we selected serial sections from 62 plaques, of which 28 were defined as fibrous plaques and 34 were defined as atheromatous plaques, according to the Athero-Express protocol and subjected these to a more laborious cell-counting method. All the MMP-12– and CD-68–positive cells were counted and the ratio was computed. The 2 methods showed a high level of correlation (*r*=0.744, *P*<0.001), and the differences between them were within the limits of agreement, as described by Bland and Altman.^24^

### Baseline Characteristics

With respect to the clinical baseline data, we found that plaques from female patients had a significantly lower percentage of MMP-12–positive macrophages compared with plaques from male patients (Table 1). In addition, plaques from patients with a previous coronary intervention or myocardial infarction had a significantly higher percentage of MMP-12–positive macrophages (Table 1).

**Figure 3.**
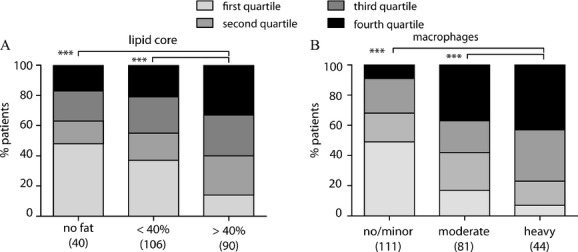
Vulnerable plaque characteristics and the percentage of patients within the 4 quartiles of MMP-12–positive macrophages. A, The amount of lipid core (abbreviated to fat) within the plaque was semiquantitatively scored. B, The amount of macrophages, also semiquantitatively scored. ***P<0.001 ordinal regression.

**Figure 4.**
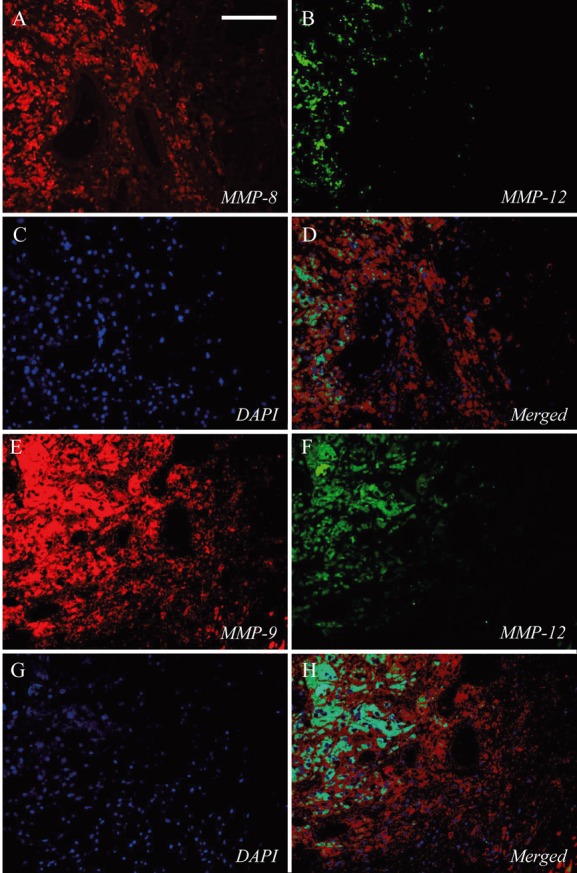
Immunohistochemical staining of adjacent segments for (A) MMP-8, (B) MMP-12, (C) 4′,6-diamidino-2-phenylindole (DAPI), and D merged and for (E) MMP-9, (F) MMP-12, (G) DAPI, and (H) merged: demonstrating colocalization of MMP-12 with MMP-8 and MMP-9.

**Figure 5.**
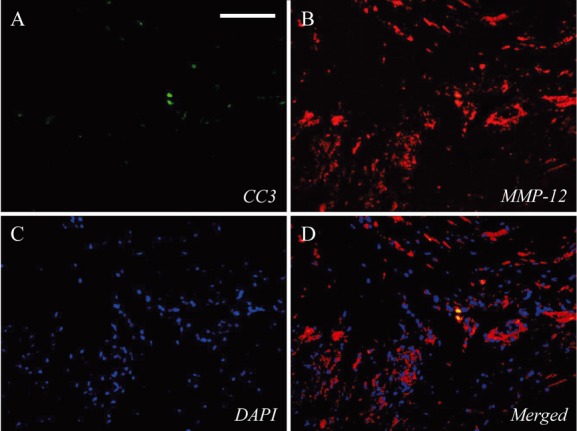
Immunohistochemical staining for (A) cleaved caspase-3 (CC3), (B) (MMP-12), (C) nuclear staining (4′,6-diamidino-2-phenylindole [DAPI]), and (D) merged, demonstrating colocalization of CC3 with MMP-12. All CC3-positive cells were also positive for MMP-12.

Based on values from all the plaques, the percentage of MMP-12–positive macrophages was significantly associated with the amount of lipid core (Figure 3, Table 2) and with the amount of macrophages, as measured both semiquantitatively (Figure 3, Table 2) and quantitatively (*r*=0.436, n=235, *P*<0.001). The prevalence of MMP-12–positive macrophages negatively correlated with the presence of SMCs (Figure 3, Table 2). We did not observe a significant relationship between MMP-12–positive macrophages and calcifications, the amount of collagen, or the presence of thrombus or intraplaque hemorrhage (Table 2). On the basis of these data, we concluded that a higher proportion of MMP-12–positive FCMs are present in highly inflamed, lipid-rich plaques with relatively reduced smooth muscle cell content. Furthermore, the MMP-12–positive FCM subpopulation significantly correlated with activity of MMP-8 (*r*=0.211, n=233, *P*=0.001), MMP-9 (*r*=0.251, n=233, *P*<0.001), and cleaved caspase-3 (*r*=0.142, n=217, *P*=0.036), a marker of apoptosis, but not with MMP-2 (*r*=0.104, n=235, *P*=0.114) measured in lysates of the segment adjacent to the culprit lesion taken from the same plaques (Table 2). Dual immunohistochemistry demonstrated that MMP-12–positive FCMs were a subpopulation of the MMP-8– and MMP-9–positive FCMs in these CEA plaques (Figure 4). Although few cleaved caspase-3–positive macrophages were found, these were all located near the necrotic core (Figure 5) and all were MMP-12 positive. This implies that all apoptotic FCMs arose from the MMP-12–positive subpopulation (Figure 5).

**Table 2.** Percentage of MMP-12 positive macrophages in relation to semi-quantitative plaque characteristics

Plaque feature	No/Minor	n	Moderate	n	Heavy	n	*P*
Calcifications	1.2 [0–20]	107	2.6 [0–38]	73	1.8 [0–22]	56	0.716

Collagen	1.2 [0–32]	56	2.2 [0–26]	128	1.9 [0–22]	52	0.610

Macrophages	0.1 [0–6]	111	11.4 [0–45]	81	13.8 [2–66]	44	*<0.001*

Smooth muscle cells	5.6 [0–27]	82	1.8 [0–34]	106	0.1 [0–15]	48	*0.036*


**Plaque feature**	**Absent**	**n**	**Present**	**n**	***P***
Lipid core > 40%	1.0 [0–17]	146	8.0 [0–43]	90	0.001

Increased vessel density (> median)*	1.8 [0–29]	113	2.9 [0–25]	110	0.775

Intra-plaque Haemorrhage	1.1 [0–23]	159	5.4 [0–32]	77	0.134

Thrombus composite	0.4 [0–23]	69	2.4 [0–25]	167	0.343

**Plaque feature**	***R***	***P***	**Plaque feature**	***R***	***P***
Macrophages (quantitatively)	0.436	< 0.001	MMP-2	0.104	0.114

Smooth Muscle Cells (quantitatively)	-0.053	0.425	MMP-8	0.211	0.001

Vessel density*	0.034	0.609	MMP-9	0.251	<0.001

			Caspase-3	0.142	0.036

Median values of MMP-12/CD68 area x 100 are provided for different plaque features, with Q1–Q3 depicted between parentheses, and the number of observations (n) in the preceding column. Quantitatively determined plaque features are depicted in the last rows, together with the correlation coefficient (Spearman) and *P*-value.

* Vessel density was not determined for 13 patients.

### Relationship With Clinical Outcome

The estimates of percentages of MMP-12–positive macrophages were not normally distributed (Figure 2E); hence, for further statistical analysis, the patients were divided into quartiles, based on the MMP-12/CD68 ratio taken for their plaque sections (Figure 2F).

**Figure 6.**
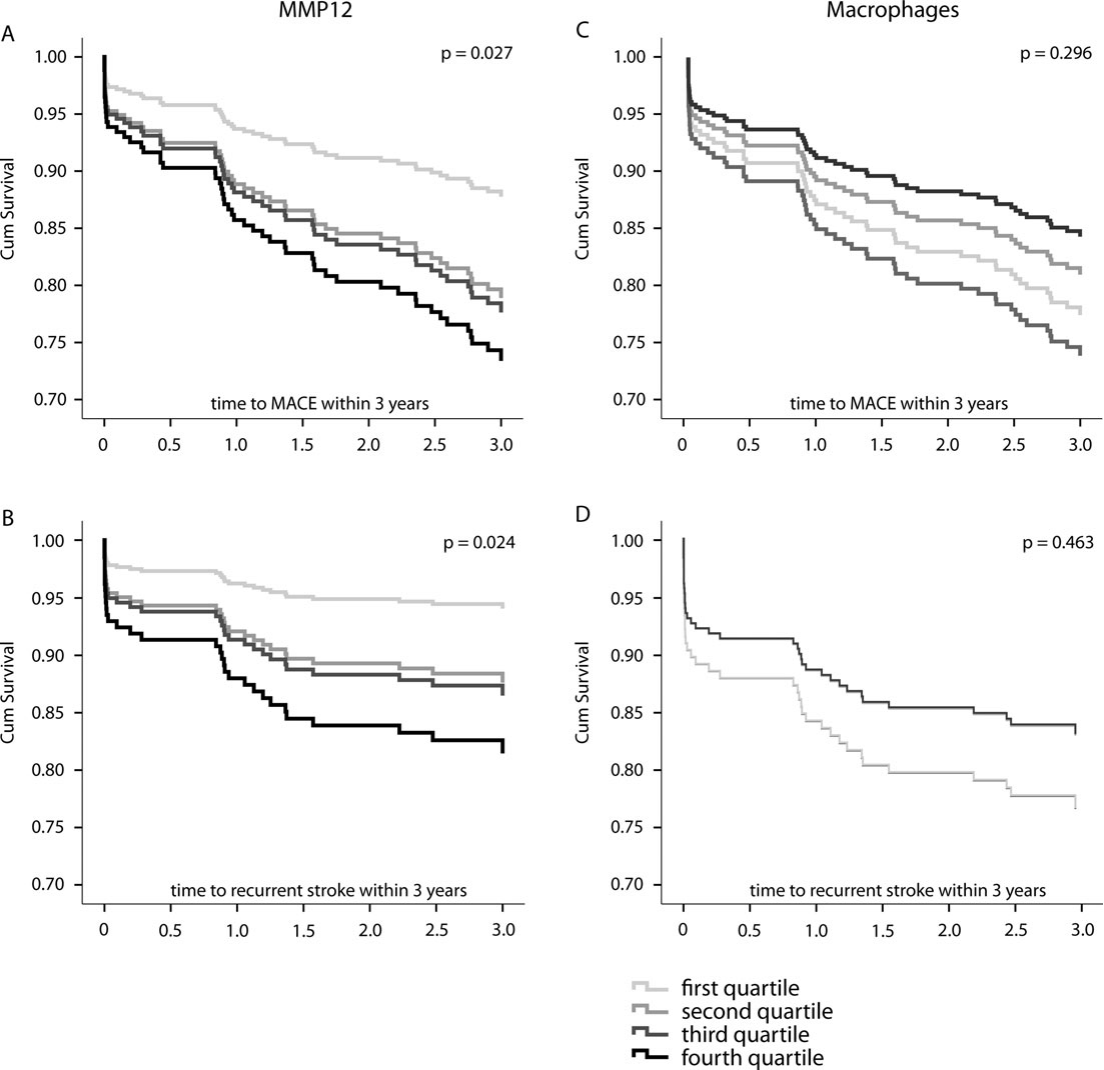
Adjusted survival curves for the 4 different quartiles, based on MMP-12/CD68 ratio. A, MACE. B, Any recurrent stroke. Adjusted survival curves for the 4 different quartiles, based on quantitatively determined macrophages: C, MACE. D, Any recurrent stroke. Adjusted *P*-values for the first vs fourth quartile are shown.

In our chosen sample, 55 (23%) patients developed an MACE during 3 years of follow-up and 27 (11%) patients sustained a stroke. The occurrence of MACEs and strokes across the 4 quartiles of MMP-12 positivity is shown in Table 3. Patients within the highest quartile of MMP-12–positive macrophages had a 2.4-fold increased risk to develop an MACE (hazard ratio, 2.4; 95% CI, 1.1 to 5.1; adjusted *P*=0.027) compared with patients within the lowest quartile (Table 3, Figure 6A). This risk increased to 3.4 for stroke (3.4; 1.2 to 9.6; *P*=0.024) (Table 3, Figure 6B). The results of univariate and multivariate analyses taking into account baseline characteristics are shown in Table 4. Importantly, although the prevalence of the MMP-12–positive subpopulation predicted adverse outcome, the total number of (CD68-positive) FCMs did not correlate with clinical outcome, as previously reported (Figure 6C and 6D).^18^

**Table 3.** Clinical Outcome and the Presence of MMP-12–Positive Macrophages

Quartile	Major End Point, n (%)	Stroke End Point, n (%)
First	**16**	**7**

Second	25	15

Third	24	14

Fourth	**31**	**20**

*P*-value (first vs fourth)	*0.027*	*0.024*

Patients were divided into quartiles based on their percentage of MMP-12–positive macrophages. The percentage of patients who developed an end point and the *P*-values adjusted for sex and age (major end point) and age (stroke) are depicted.

**Table 4.** Univariate and Multivariate Cox Regression Analyses of Risk Factors and MMP-12 Quartiles

	Univariate Cox Regression		Multivariate Cox Regression (Enter)	
		
Clinical Characteristic	*P*	HR (CI)	*P*	HR (CI)
Major end point				

Sex	0.039	2.309 [1.045 to 5.104]	*0.108*	

Age	<0.001	1.080 [1.044 to 1.118]	<*0.001*	1.086 [1.047 to 1.126]

Smoking*	0.996			

Diabetes	0.224			

Hypertension	0.987			

Hypercholesterolemia	0.521			

Family history of heart disease†	0.452			

GFR‡	0.217			

MMP-12/CD68 ratio (quartiles)	0.242			

Fourth vs first quartile	0.042	2.182 [1.030 to 4.624]	*0.027*	2.369 [1.104 to 5.083]

Stroke end point				

Sex	0.448			

Age	0.002	1.074 [1.027 to 1.123]	*0.001*	1.080 [1.031 to 1.132]

Smoking*	0.462			

Diabetes	0.388			

Hypertension	0.344			

Hypercholesterolemia	0.420			

Family history of heart disease†	0.094			

GFR‡	0.835			

MMP-12 ratio (quartiles)	0.222			

Fourth vs first quartile	0.036	3.049 [1.074 to 8.659]	*0.024*	3.358 [1.176 to 9.583]

*P*-values and hazard ratios (HRs), with 95% CIs depicted between brackets. Risk factors with a *P*-value <0.1 were included in the multivariate Cox regression (enter) model to correct for potential confounding.

* The smoking status of 2 patients could not be retrieved.

† The family history of 17 patients could not be retrieved.

‡ The glomerular filtration rate (GFR) of 1 patient could not be retrieved.

## Discussion

### Main Findings

We are, to our knowledge, the first to report that presence of a specific subset of FCMs in plaques predicts adverse patient outcome. Using a relatively large cohort of patients, we show that the proportion of MMP-12–positive FCMs is higher in CEA tissues from patients who sustained adverse clinical outcomes during 3 years of follow-up.

### MMP-12 and Vulnerable Plaque Features

Animal and human studies have demonstrated that MMP-12 is absent in healthy arteries, minimally present in early lesions, and strongly expressed in advanced atherosclerotic plaques.^8,9,12–14^ However, the relationship between the presence of MMP-12–positive macrophages and markers of plaque vulnerability has remained uncertain. To quantify the percentage of MMP-12–positive macrophages within a relatively large number of samples, we developed a new, semiautomated method and validated this against a more laborious manual counting method. The percentage of MMP-12–positive macrophages was associated with vulnerable plaque features, namely the presence of large lipid cores and abundance of macrophages, and negatively with the presence of smooth muscle cells, which is considered a feature of a more stable plaque phenotype.^25^ The percentage of MMP-12–positive macrophages was not significantly related to other histological features of vulnerable plaques such as the presence of thrombus or intraplaque hemorrhage, perhaps as a result of inadequate statistical power.

The hypothesis that MMP-12 is causal in, rather than merely a marker of, plaque instability is borne out by a substantial body of experimental animal research. For example, work from our group and another^5,10^ found that MMP-12 knockout in apolipoprotein E–deficient mice reduces lesion progression, macrophage infiltration, lipid core formation, calcification, degradation of elastic lamellae, and the development of buried fibrous layers that may reflect silent plaque ruptures. Work in transgenic rabbits also shows that active MMP-12 can promote the development of advanced plaques.^8^ In the present study, we showed that macrophages positive for MMP-12, which is a metalloelastase, were also positive for a collagenase, MMP-8, and a gelatinase, MMP-9, which we have previously reported are strongly related with a more vulnerable plaque phenotype.^20,21^ Together, these 3 MMPs have a broad specificity for extracellular matrix components, which may add to their destructive potential. Apart from destruction of the extracellular matrix, MMPs can also cleave nonmatrix components, leading to modulation of the migration, proliferation, and apoptosis of vascular cells.^26^ In this context, it is particularly interesting to note the recently reported ability of MMP-12 to promote apoptosis of mouse and human macrophages.^11^ This may explain why, in our study, we found that cleaved caspase-3 levels correlated and colocalized with MMP-12–positive macrophages. Macrophage apoptosis contributes to lipid core formation,^27^ which, as noted, was also associated with MMP-12 positivity in our study. Our results, therefore, at least show that MMP-12 may be causative in plaque macrophage apoptosis, promoting plaque progression and instability by augmenting lipid core formation.

### MMP-12 and Clinical Parameters

Compared with female patients, male patients are more prone to develop cardiovascular events during life and are at increased risk for recurrent cardiovascular events,^2,28^ as also shown in our present study (Table 4). Furthermore, we observed a significantly lower prevalence of MMP-12–positive FCMs within the carotid plaques of female patients (Table 1). We have previously reported that female patients have more stable atherosclerotic plaques with less lipid and macrophages, more smooth muscle cells, and higher collagen content.^29^ In addition, levels of interleukin-8 and MMP-8 activity were found to be lower. From our present data, lowered prevalence of MMP-12–positive macrophages appears to be another factor possibly related to the protective effect of female sex on atherosclerosis.

We clearly demonstrated that the proportion of MMP-12–positive FCMs was greater in patients who had undergone CEA and developed secondary cardiovascular events during follow-up. Patients within the fourth quartile of MMP-12–positive macrophages had, compared with patients within the lowest quartile, a 2.4-times increased risk of an MACE and a 3.4-times increased risk of a stroke. Although the number of strokes was low and the observed increased risk has to be interpreted with caution, these differences persisted after correction for age and sex. The differences in outcome were mainly apparent between patients within the first quartile, whose plaques had no MMP-12–positive FCMs, and those within the fourth quartile, for whom >25% of FCMs were positive for MMP-12 (Figure 2F, Table 3).

Atherosclerosis is a systemic disease^30^ and plaque composition is related between different arterial territories in a given patient.^31^ It is not surprising, therefore, that atherosclerotic plaques sampled from one location might contain markers predictive for future cardiovascular events in other parts of the arterial tree. We previously reported that several proteins expressed in local plaques and some histological features, including intraplaque hemorrhage and microvessel density, can provide prognostic information for the occurrence of events in other vascular territories.^17,19,20^ Interestingly, however, we reported that the total amount of CD68-positive FCMs in CEA plaques is not associated with adverse clinical outcome,^18^ as confirmed in our study (Figure 6C and 6D). Hence, our new data showing that the presence of a specific FCM subtype expressing MMP-12 is associated with adverse clinical outcome are not confounded by its relationship to total numbers of FCMs.

## Conclusions

Our results show for the first time that a subset of macrophages expressing MMP-12 in human atherosclerotic plaques are associated with vulnerable plaque characteristics and adverse clinical outcome. This new information adds to our understanding of the systemic nature of atherosclerotic disease. It also builds on a substantial body of experimental evidence from animals to emphasize the potential of MMP-12 as therapeutic target. The ongoing development and refinement of MMP-12–specific inhibitors amplify the implications of our study for plaque stabilization therapy. Furthermore, although clearly beyond the scope of the present study, MMP-12 activity might form the basis of a method to identify vulnerable plaques. For example, several studies have established the practicality of imaging MMP activity in plaques.^32,33^ Hence, a technique based on visualizing MMP-12 protein or its activity using a selective substrate might be possible.
